# Chromatin regulators in retinoblastoma: Biological roles and therapeutic applications

**DOI:** 10.1002/jcp.30022

**Published:** 2020-08-25

**Authors:** Chunsik Lee, Jong Kyong Kim

**Affiliations:** ^1^ State Key Laboratory of Ophthalmology, Zhongshan Ophthalmic Center Sun Yat‐sen University Guangzhou China

**Keywords:** chromatin, epigenetic regulator, ocular tumor, retinoblastoma, retinogenesis, targeted therapy

## Abstract

Retinoblastoma (RB) is a pediatric ocular tumor mostly occurring due to the biallelic loss of *RB1* gene in the developing retina. Early studies of genomic aberrations in RB have provided a valuable insight into how RB can progress following the tumor‐initiating *RB1* mutations and have established a notion that inactivation of *RB1* gene is critical to initiate RB but this causative genetic lesion alone is not sufficient for malignant progression. With the advent of high‐throughput sequencing technologies, we now have access to the comprehensive genomic and epigenetic landscape of RB and have come to appreciate that RB tumorigenesis requires both genetic and epigenetic alterations that might be directly or indirectly driven by *RB1* loss. This integrative perspective on RB tumorigenesis has inspired research efforts to better understand the types and functions of epigenetic mechanisms contributing to RB development, leading to the identification of multiple epigenetic regulators misregulated in RB in recent years. A complete understanding of the intricate network of genetic and epigenetic factors in modulation of gene expression during RB tumorigenesis remains a major challenge but would be crucial to translate these findings into therapeutic interventions. In this review, we will provide an overview of chromatin regulators identified to be misregulated in human RB among the numerous epigenetic factors implicated in RB development. For a subset of these chromatin regulators, recent findings on their functions in RB development and potential therapeutic applications are discussed.

## Graphical abstract

In retinoblastoma (RB) cells, E2F activity remains unconstrained and promotes expression of cell cycle genes and chromatin regulators, leading to uncontrolled proliferation and aberrant alterations in epigenetic landscape and gene expression. These downstream effects of deregulated E2F activity are postulated to drive RB tumorigenesis. In this review, recent findings on the functions of misregulated chromatin regulators in RB are discussed.

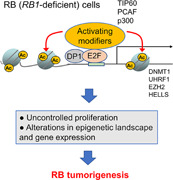

## 1 INTRODUCTION

Retinoblastoma (RB) is a rare type of childhood cancer but is a major intraocular malignancy affecting young children with a global incidence rate of 1 in 16,000–18,000 live births per year. RB develops as a result of biallelic inactivation of *RB1* tumor suppressor gene in the developing retina for a vast majority of cases (Dimaras et al., 2015). As *RB1* gene mutation in either germline or somatic cells is required to initiate RB development and other genomic changes have also been found in primary tumors, RB has been considered as a genetic disease and early studies have focused on identification of additional genetic lesions that might cooperate with the tumor‐initiating *RB1* mutations to drive malignant progression of RB. These efforts have identified several recurrent chromosomal abnormalities in RB along with some candidate oncogenes and tumor suppressor genes within the identified genomic regions (Corson & Gallie, 2007). Later, whole‐genome sequencing of four RB tumors and their paired germline DNA samples has revealed that RB genomes are more stable than previously thought and very few genetic lesions are required for tumor progression after *RB1* inactivation. Moreover, an integrative epigenetic analysis of RB from the same study demonstrated that multiple cancer pathways are deregulated by epigenetic mechanisms, suggesting that epigenetic dysregulation might act as a critical driver of RB tumorigenesis in the absence of other major genetic lesions apart from *RB1* mutations (Zhang et al., 2012). Another independent study in a large RB cohort has shown that genomic alterations as single nucleotide variants are rare in RB and somatic copy number alterations are more common, validating the presence of the recurrent chromosomal alterations identified previously (Kooi et al., 2016). Now, it is well accepted that RB tumorigenesis and further progression require additional genetic and epigenetic alterations following *RB1* inactivation, and epigenetic dysregulation in RB has been observed for nearly all areas of epigenetics including DNA methylation, histone modifications, and noncoding RNAs as exemplified by promoter hypermethylation of tumor suppressor genes, activating histone modifications at the promoter of cancer pathway genes such as *SYK*, and aberrant downregulation of microRNAs with tumor suppressive roles (Benavente & Dyer, 2015; U. Singh, Malik, Goswami, Shukla, & Kaur, 2016; Theriault, Dimaras, Gallie, & Corson, 2014). Some of the epigenetic dysregulation reported in RB will be further described later in the section where biological functions of chromatin regulators in RB are discussed. Interestingly, pan‐cancer genomic analyses from 24 types of childhood cancers have shown that overall somatic coding mutation frequencies of pediatric cancers are much lower than those of adult cancers, with the mutation frequency of RB ranked as the third lowest in the pan‐cancer cohort (Grobner et al., 2018). Furthermore, sequencing for most of known epigenetic regulators in over 1,000 pediatric cancers comprising 21 distinct cancer types demonstrated that many of these childhood cancers have a varied frequency of somatic mutations in almost every class of epigenetic regulators although some of the cancer types such as low‐grade glioma and RB display a near‐complete absence of mutations in epigenetic regulators (Huether et al., 2014). These systemic analyses for the landscape of somatic mutations across pediatric cancers consistently demonstrate that causative genetic lesions underlying the tumor initiation can be identified despite the low mutation rates in the genomes, and suggest that other epigenetic events might play key roles in the development of these tumors.

RB tumorigenesis has been understood in the context of retinal development as malignant transformation initiated by *RB1* inactivation is thought to disrupt the gene regulatory network of proliferation and differentiation during retinogenesis and consequently drive RB development. Along with gene perturbation approaches in mice, genome‐wide epigenetic profiling in whole retina or purified retinal cell types over different developmental stages has provided valuable insights into the epigenetic landscape in the retina and its relation to retinogenesis and RB development when combined with the corresponding transcriptomic analyses (Aldiri et al., 2017; Corso‐Diaz, Jaeger, Chaitankar, & Swaroop, 2018). Indeed, this integrative approach has suggested that murine and human RB epigenomes resemble those of normal retina at the developmental stage when retinal progenitors switch from neurogenic to terminal patterns of cell division, and that epigenetically regulated genes during retinal development are highly likely to be perturbed in RB (Aldiri et al., 2017). Therefore, temporal and cell type‐specific expression of transcription factors and epigenetic modifiers would be critical to ensure the stringent control of gene expression and thereby support normal retinal differentiation and cellular homeostasis. Given the dynamic changes in the epigenome during retinal development and accumulating evidence on epigenetic aberrations in human RB tumors, RB cells are predicted to have high cellular plasticity which might contribute to their undifferentiated phenotypes or dedifferentiation. Thus, inhibiting epigenetic regulators mediating these effects may suppress unfavorable cellular reprogramming and tumor progression. Aside from this notion, a recent review has presented a new model of cell type‐specific disease susceptibility termed cellular pliancy, which highlights how epigenome organization and its alterations would define cellular pliancy to prime retinal disease conditions (Dyer, 2016). Altogether, these concepts emphasize the importance of epigenetic regulation in retinal development and diseases, and present a need to advance our incomplete understandings on the subject to better cope with retinal diseases including retinoblastoma.

For the past decade, gene expression profiling in human RB tissues has identified numerous chromatin regulators misregulated in the tumors, but the impact of these chromatin regulators on RB tumorigenesis and malignant progression has just begun to be understood. This review will provide an overview of these misregulated chromatin regulators identified in human RB with possible contributions of E2F family proteins to epigenetic aberrations observed in RB. Then, we will focus on select chromatin regulators and recent findings on their functions in RB development. For a subset of these chromatin regulators, potential therapeutic applications are also discussed.

## 2 ABERRANT EXPRESSION OF CHROMATIN REGULATORS AND IMPLICATIONS OF E2F FAMILY PROTEINS IN EPIGENETIC DYSREGULATION IN RB

Understanding of the molecular mechanisms driving RB tumor progression following tumor‐initiating mutations in *RB1* gene has been a subject of intense research since the discovery of *RB1* tumor suppressor gene and its alterations as causative genetic lesions for RB development. To get a clue on this fundamental question, several gene expression profiling studies were conducted to identify differentially expressed genes in human RB tumors as compared to adjacent normal retina or independent normal retinal tissues (Chakraborty et al., 2007; Ganguly & Shields, 2010; Kapatai et al., 2013; Rajasekaran et al., 2019) or to determine gene expression signatures in large cohorts of primary RB tumors to obtain mechanistic insights into tumor progression and cells of origin (Kooi et al., 2015; McEvoy et al., 2011). Gene ontology analyses from these studies indicated that genes involved in chromatin/nucleosome assembly and organization are highly enriched in human RB as compared to normal retina. Consistent with an increasing role of epigenetic dysregulation in RB tumorigenesis and progression, a number of chromatin modifiers and chromatin‐associating proteins were found to be upregulated in human RB (Table 1). Many of these misregulated chromatin regulators are fetal proteins that are normally expressed only during retinal development, thus their expression is not detected in terminally differentiated retina (Benavente et al., 2014; Chau et al., 2003; Kan et al., 2017; Khan et al., 2015; Qu et al., 2010; Zocchi et al., 2020). However, RB tumors display a varying degree of overexpression for these epigenetic regulators. This observation supports a notion that RB is a developmental tumor associated with perturbations in spatiotemporal control of gene expression required for normal retinal development, and epigenetic dysregulation occurring during this process is highly likely to be involved in tumor initiation and malignant progression.

**Table 1:** Differentially expressed chromatin regulators in human RB relative to normal retina

Functional category	Gene	Name	Expression	References
DNA methylation	*DNMT1*	DNA methyltransferase 1	Up	Ganguly and Shields (2010); Rajasekaran et al. (2019); Qu et al. (2010)
	*DNMT3A*	DNA methyltransferase 3A	Up	Rajasekaran et al. (2019); Qu et al. (2010)
	*DNMT3B*	DNA methyltransferase 3B	Up	Ganguly and Shields (2010); Rajasekaran et al. (2019); Qu et al. (2010)
DNA demethylation	*TET1*	Tet methylcytosine dioxygenase 1	Up	Ganguly and Shields (2010); Rajasekaran et al. (2019)
	*TET3*	Tet methylcytosine dioxygenase 3	Up	Ganguly and Shields (2010); Rajasekaran et al. (2019)
Polycomb group	*EZH2*	Enhancer of zeste 2 polycomb repressive complex 2 subunit	Up	Ganguly and Shields (2010); Kapatai et al. (2013); Khan et al. (2015); Rajasekaran et al. (2019)
	*SUZ12*	SUZ12 polycomb repressive complex 2 subunit	Up	Chakraborty et al. (2007); Ganguly and Shields (2010)
	*BMI1*	BMI1 proto‐oncogene, polycomb ring finger	Up	Kapatai et al. (2013); R. Ren et al. (2013)
	*PCGF6*	Polycomb group ring finger 6	Up	Ganguly and Shields (2010); Kapatai et al. (2013); Rajasekaran et al. (2019)
	*JARID2*	Jumonji and AT‐rich interaction domain containing 2	Up	Ganguly and Shields (2010); Kapatai et al. (2013)
	*CBX2*	Chromobox 2	Up	Ganguly and Shields (2010); Rajasekaran et al. (2019)
	*CBX6*	Chromobox 6	Down	Ganguly and Shields (2010); Kapatai et al. (2013); Rajasekaran et al. (2019)
	*CBX7*	Chromobox 7	Down	Kapatai et al. (2013); Rajasekaran et al. (2019)
Histone modification	*EHMT2*	Euchromatic histone lysine methyltransferase 2	Up	Ganguly and Shields (2010); Kapatai et al. (2013); Rajasekaran et al. (2019)
	*HDAC2*	Histone deacetylase 2	Up	Ganguly and Shields (2010); Rajasekaran et al. (2019)
	*SUV39H2*	Suppressor of variegation 3–9 homolog 2	Up	Ganguly and Shields (2010); Rajasekaran et al. (2019)
	*SMYD3*	SET and MYND domain containing 3	Up	Ganguly and Shields (2010); Rajasekaran et al. (2019)
	KDM1A	Lysine demethylase 1A	Up	Ganguly and Shields (2010); Rajasekaran et al. (2019)
Chromatin remodeling	*HELLS*	Helicase, lymphoid specific	Up	Ganguly and Shields (2010); Kapatai et al. (2013); Rajasekaran et al. (2019)
	*SMARCC1*	SWI/SNF‐related, matrix‐associated actin‐dependent regulator of chromatin, subfamily C, member 1	Up	Ganguly and Shields (2010); Rajasekaran et al. (2019)
	*SMARCAD1*	SWI/SNF‐related, matrix‐associated actin‐dependent regulator of chromatin, subfamily A, containing DEAD/H box 1	Up	Ganguly and Shields (2010); Rajasekaran et al. (2019)
Chromatin‐associating proteins	*UHRF1*	Ubiquitin‐like with PHD and ring finger domains 1	Up	Ganguly and Shields (2010); Kapatai et al. (2013); Kan et al. (2017); Rajasekaran et al. (2019)
	*HMGA1*	High mobility group AT‐hook 1	Up	Ganguly and Shields (2010); Kapatai et al. (2013); Rajasekaran et al. (2019)
	*HMGA2*	High mobility group AT‐hook 2	Up	Ganguly and Shields (2010); Kapatai et al. (2013); Rajasekaran et al. (2019)
	*HMGB2*	High mobility group box 2	Up	Ganguly and Shields (2010); Rajasekaran et al. (2019)
	*HMGB3*	High mobility group box 3	Up	Ganguly and Shields (2010); Kapatai et al. (2013); Rajasekaran et al. (2019)
	*CHAF1B*	Chromatin assembly factor 1 subunit B	Up	Ganguly and Shields (2010); Kapatai et al. (2013); Rajasekaran et al. (2019)
	*CBX5*	Chromobox 5	Up	Ganguly and Shields (2010); Kapatai et al. (2013); Rajasekaran et al. (2019)
	*CBX1*	Chromobox 1	Up	Ganguly and Shields (2010); Rajasekaran et al. (2019)
	*CBX3*	Chromobox 3	Up	Ganguly and Shields (2010); Rajasekaran et al. (2019)
	*MBD2*	Methyl‐CpG binding domain protein 2	Down	Ganguly and Shields (2010); Rajasekaran et al. (2019)

*Note*: Differential expression detected for indicated chromatin regulators in primary human RB tumors relative to normal retina by gene expression profiling or immunohistochemistry. Due to tumor sample heterogeneity and variance in cohort size among the cited references, chromatin regulators that are redundantly found to be misregulated in multiple independent studies are listed.

**Figure 1:**
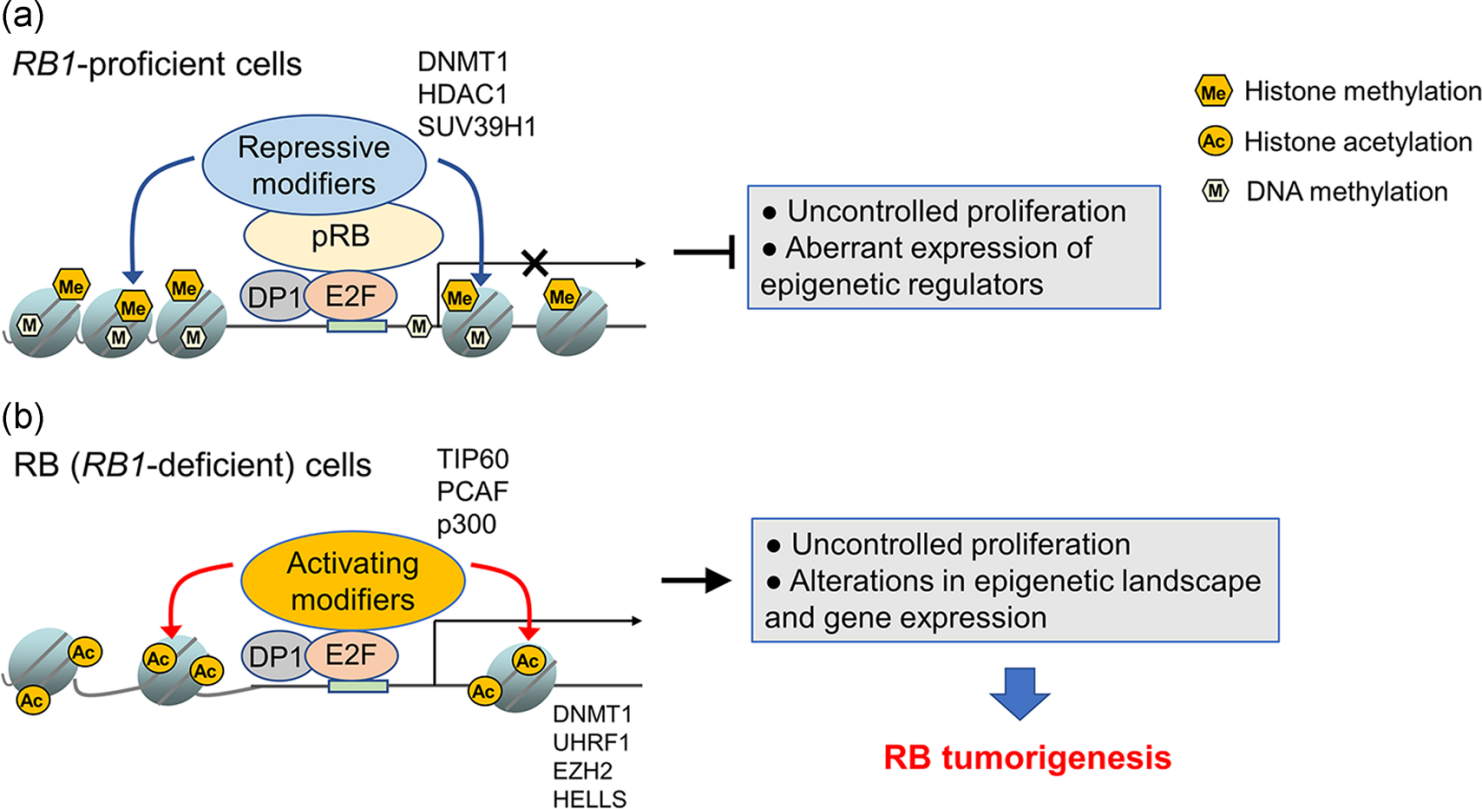
*RB1* loss drives RB tumorigenesis through deregulated E2F activity. (a) In *RB1*‐proficient cells, functional pRB directly inhibits the transactivation domain of E2Fs and also keeps E2F target genes in check by recruiting repressive chromatin modifiers such as DNMT1, HDAC1, and SUV39H1 at the target gene promoters, thereby restraining uncontrolled proliferation and aberrant expression of epigenetic regulators. (b) In RB cells lacking functional pRB, E2F activity remains unconstrained and promotes transcription of cell cycle genes and epigenetic regulators by directly recruiting activating chromatin modifiers such as histone acetyltransferases (TIP60, PCAF, p300) to target promoters, leading to uncontrolled proliferation as well as alterations in epigenetic landscape and gene expression due to aberrant expression of chromatin regulators. These downstream effects of deregulated E2F activity are postulated to drive RB tumorigenesis. DNMT1, DNA methyltransferase 1; HDAC1, histone deacetylase 1; pRB, RB protein; RB, retinoblastoma

Interestingly, many of misregulated chromatin regulators in RB (including ubiquitin‐like with PHD and RING finger domains 1 [UHRF1], DNA methyltransferase 1 [DNMT1], enhancer of zeste homolog 2 [EZH2], B lymphoma Mo‐MLV insertion region 1 [BMI1], and HELLS) have been found to be direct transcriptional targets upregulated by E2F family transcription factors in various cell types (Bracken et al., 2003; Magri et al., 2014; Nowak et al., 2006; Unoki, 2011; Zocchi et al., 2020), coupling *RB1* inactivation‐induced deregulation of E2F activity to protumorigenic effects manifested by alterations of chromatin regulators (Figure 1). In addition to the direct transcriptional role in the upregulation of chromatin regulators, E2F family proteins may play more active and diverse roles in epigenetic dysregulation in RB. Of note, E2F family proteins themselves can recruit several chromatin modifiers such as TIP60 acetyltransferase complex and alter local chromatin structure and gene expression (Blais & Dynlacht, 2007; Taubert et al., 2004). Moreover, an unbiased location analysis of E2F1‐binding sites in HeLa cells demonstrated that a large fraction (25–35%) of genes in human cancer cells have E2F1 binding on their promoters and the vast majority of these actual E2F1‐binding sites in cells lack the canonical E2F1‐binding motifs (Bieda, Xu, Singer, Green, & Farnham, 2006). Not only in gene promoters but also in noncoding genomic regions enriched in repetitive sequences, RB protein (pRB) binding was detected and its association with the repeats was diminished when a CDK‐resistant pRB‐E2F1 interaction was disrupted, implying that E2F1 may also be recruited to repeat sequences although the recognition mechanism by E2F1 is unknown (Ishak et al., 2016). These observations suggest that actual E2F‐binding sites would outnumber the prediction based on the consensus E2F‐binding motifs in the genome and the deregulated E2F activity in RB cells may exert more profound effects on epigenetic dysregulation in cooperation with chromatin modifiers or other regulatory proteins in the complexes that assemble independently of putative E2F‐binding sequences. To date, there have been no studies profiling E2F‐binding sites genome‐wide in RB cells; however, it becomes evident that deregulated E2F family proteins in RB cells are implicated in the misregulation of chromatin regulators and possibly contribute to aberrant epigenetic landscape in RB. Notably, chromatin regulators *Hells* and *Uhrf1* were identified to be expressed in murine RB tumors, but their expression was abrogated in *E2f1* or *E2f3‐*deficient background in the same murine RB model with a concomitant rescue of the tumor development phenotype (Benavente et al., 2014). This finding illustrates that deregulated E2F family proteins may play a pivotal role in RB tumorigenesis through epigenetic regulation in addition to their canonical function in promoting uncontrolled cell proliferation (Figure 1).

Of note, pRB itself is implicated in regulation of chromatin structure and gene expression by associating with various chromatin regulators, including DNA and histone modifiers, chromatin remodelers, and chromatin‐binding proteins. These epigenetic regulators have been found to be tethered by pRB or pRB/E2F, acting locally at the gene promoters regulated by E2F or other transcription factors and also playing global regulatory roles in large‐scale chromatin structures such as pericentromeric heterochromatin (Talluri & Dick, 2012; Uchida, 2016). Based on the epigenetic dysregulation observed in RB, understanding the normal functions of pRB in chromatin regulation may give some useful hints on how *RB1* inactivation may influence the overall epigenetic landscape in human RB. Gene expression studies from multiple models of *RB1* deletion have identified a highly conserved signature of genes which are implicated in epigenetic regulation and DNA damage response/repair in addition to the canonical pRB pathway targets involved in cell cycle progression and DNA replication (Knudsen, Pruitt, Hershberger, Witkiewicz, & Goodrich, 2019). All these findings bear potential relevance to understanding of human RB pathogenesis although it has yet to be determined to what extent these findings can be extrapolated to the case of RB which lacks functional pRB from the initiation of tumors. In particular, it would be interesting to investigate whether these pRB functions in normal chromatin regulation would require the presence of E2Fs as is the case with pRB/EZH2 complex‐mediated silencing of repetitive sequences that was reported to require a cell cycle‐independent interaction between pRB and E2F1 (Ishak et al., 2016). If this is the case for many other pRB‐mediated chromatin regulation events, it would be important to determine how the deregulated E2F activity in tumors with *RB1* loss would impact the tumor epigenomes.

## 3 BIOLOGICAL ROLES OF MISREGULATED CHROMATIN REGULATORS IN RB DEVELOPMENT

Although a plethora of chromatin regulators have been identified to be misregulated in human RB, gene expression analyses with human RB tumors are often limited by tumor sample heterogeneity, small cohort size, and lack of control retina for comparative analysis. Therefore, in this section, we select a few chromatin regulators which are recurrently found to be misregulated in several independent studies and discuss their biological functions in the context of RB development.

### 3.1 DNA methyltransferase

The first evidence of epigenetic regulation involved in RB tumorigenesis came from an early finding that a CpG island (CpG 106) overlapping the promoter and Exon 1 of the *RB1* gene is methylated in a subset of tumors (Greger, Passarge, Hopping, Messmer, & Horsthemke, 1989). Since this initial report on DNA methylation as a factor of epigenetic regulation in RB development, promoter hypermethylation has been documented for several other tumor suppressor genes in addition to *RB1*, including *RASSF1A, MGMT*, and *CDKN2A* (Choy et al., 2005; Harada et al., 2002; Indovina et al., 2010; Livide et al., 2012). As in other cancers, global DNA hypomethylation was also observed in human RB genomes although it appeared to be modest as compared to normal retina (Kan et al., 2017). Therefore, human RB methylomes display similar aberrations of DNA methylation found in many cancers (Liang & Weisenberger, 2017), and DNMT family enzymes are predicted to be at the center of this dysregulation. Indeed, an immunohistochemical analysis of six normal retina and 62 RB tissues revealed frequent overexpression of DNMT1, DNMT3A, and DNMT3B in RB whereas these proteins were not detected in normal retina (Qu et al., 2010). Furthermore, expression of DNMT1 and DNMT3A was significantly higher in poorly differentiated RB than in well‐differentiated tumors while high DNMT1 expression was associated with invasive RB tumors (Qu et al., 2010). Since DNMT1 has been identified as a transcriptional target of E2F1 in mammalian cells (Magri et al., 2014), its overexpression in human RB may not be surprising, but the regulatory mode of DNMT1 on DNA methylation in RB cells has turned out to deviate from a prevailing prediction. First, DNA methylation maintenance by DNMT1 in RB cells was not dependent on UHRF1, an epigenetic regulator required for faithful inheritance of DNA methylation through cell divisions by recruiting DNMT1 to replication foci (Bostick et al., 2007; Sharif et al., 2007). Second, nuclear localization of DNMT1 in S phase RB cells showed a diffuse pattern with a very few distinct foci formed in nuclei even in the presence of intact endogenous UHRF1 and only part of replication foci colocalized with DNMT1 foci (Kan et al., 2017). These results indicate that the regulation mode of maintenance methylation in RB cells is different from that of most cancer cells. Currently, it remains unknown how RB cells show this unexpected difference in DNA methylation regulation, but the canonical function of DNMT1 in maintenance methylation appears to be preserved in RB cells as treatment of 5‐azacytidine (DNMT inhibitor) was shown to induce global hypomethylation and re‐expression of *EPCAM* gene by promoter demethylation in Y79 cells (Kan et al., 2017). In this study, global methylation levels were also compared between early onset and late‐onset murine RB tumors to evaluate if global DNA hypomethylation is a critical early mechanism driving RB tumorigenesis as previously proposed in a model of hepatocellular carcinoma (Mudbhary et al., 2014). However, there was no significant change in global methylation levels all across the mouse tumors regardless of the tumor onset time, implying that global methylation changes may not contribute to RB tumorigenesis (Kan et al., 2017). Nevertheless, pharmacological inhibition of DNMTs in RB cells may have some therapeutic efficacy in combination with other agents. For example, a previous study demonstrated that RB cells are intrinsically resistant to death receptor (DR)‐mediated apoptosis due to a deficiency of caspase‐8 expression caused by DNA hypermethylation and that 5‐azacytidine treatment restores the caspase‐8 expression and sensitivity to DR‐mediated apoptosis partially (Poulaki et al., 2005).

Collectively, there are a few key observations pointing to potential roles of DNMTs in RB development such as their frequent overexpression in advanced RB tumors and promoter hypermethylation of tumor suppressor genes. However, further understanding of how DNMTs mediate aberrant DNA methylation at both bulk genome and individual gene loci would be required for an assessment of therapeutic applications targeting DNA methylation in RB.

### 3.2 Ubiquitin‐like with PHD and RING finger domains 1

As briefly stated in the previous section, UHRF1 is an epigenetic regulator for DNA methylation and histone modifications expressed in proliferative cells and tissues (Bronner, Krifa, & Mousli, 2013). In cancer cells, UHRF1 is frequently overexpressed constitutively and known to promote tumor development by introducing changes in DNA and histone modifications via recruitment of various chromatin modifiers and thereby altering gene expression (Alhosin et al., 2016; Unoki, 2011). UHRF1 in RB development was first described in a study where genetic disruption of *E2f1* or *E2f3* in a murine RB model was found to abrogate tumor development, and searching for the candidate genes responsible for this phenotype rescue led to the identification of *Hells* and *Uhrf1* as potential epigenetic factors involved in RB tumor progression (Benavente et al., 2014). In that study, UHRF1 depletion in RB cells was shown to reduce the final size of orthotopic xenograft tumors although both UHRF1‐depleted tumors and control xenografts appeared to grow at a similar rate.

**Figure 2:**
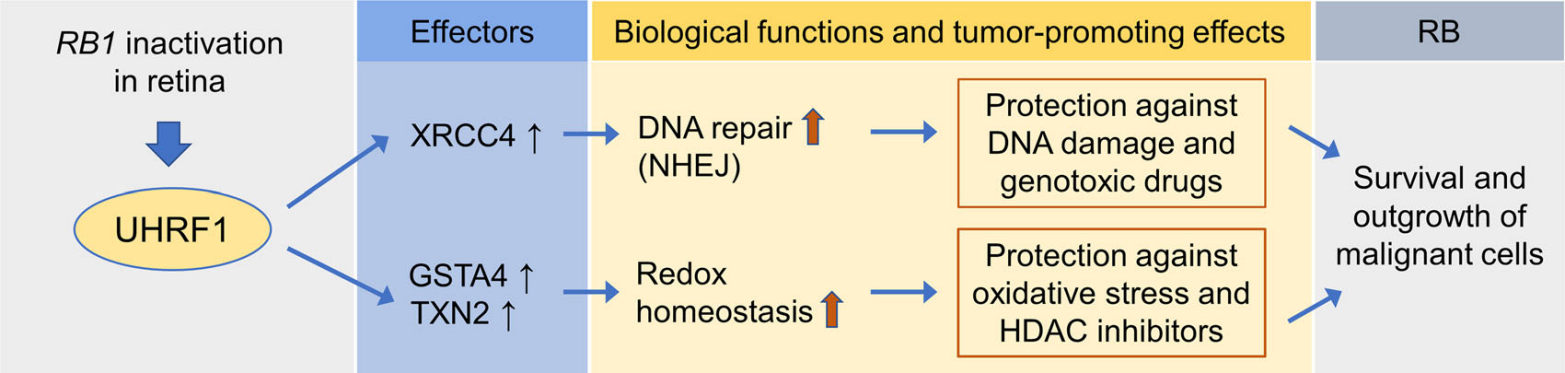
Model of UHRF1‐mediated tumor promotion in RB. Inactivation of *RB1* gene in the developing retina induces aberrant expression of UHRF1. The highly expressed UHRF1 upregulates downstream effectors implicated in DNA repair and redox homeostasis, which provides the cells with a better capacity to cope with endogenous DNA damage and oxidative stress which might arise during tumorigenesis. In addition, the enhancement of cellular stress‐managing capacity driven by UHRF1 expression also contributes to resistance against genotoxic drugs and HDAC inhibitors, endowing the cells with a selective advantage to evade apoptosis and thereby promoting survival and outgrowth of malignant tumor cells. HDAC1, histone deacetylase 1; NHEJ, nonhomologous end‐joining; RB, retinoblastoma; UHRF1, ubiquitin‐like with PHD and RING finger domains 1

Given the well‐known role of UHRF1 in regulation of DNA methylation in both normal and cancer cells, functions of UHRF1 in the establishment and maintenance of RB methylomes were investigated using UHRF1‐knockdown Y79 cells and two murine RB models. Unexpectedly, UHRF1 downmodulation in RB cells exerted very minor effects on the pre‐existing methylation patterns. Moreover, there were no significant correlations between UHRF1 expression and global methylation levels in both premalignant neonatal retina and RB tumors in two different mouse models of RB (Kan et al., 2017). A genome‐wide methylation analysis with whole retina tissues has limitations in drawing a definitive conclusion as the whole retina has many other cell types in addition to the RB‐initiating retinal cells. However, combined experimental evidence in cell lines and mouse models along with the unusual DNMT biology observed in RB cells suggest that tumor‐promoting functions of UHRF1 in RB are largely independent of its role in DNA methylation and may involve other epigenetic mechanisms. As in many other cancer cells, human RB cell lines including Y79 and Weri‐Rb1 display high expression of UHRF1, at least partially by E2F‐mediated transcriptional upregulation. However, unlike many cancer cells, UHRF1 knockdown in Y79 cells results in no gross proliferation defects except for a modest increase in apoptosis while UHRF1 depletion in Weri‐Rb1 cells has shown to induce appreciable cell cycle arrest and some increase in apoptosis via inhibition of the phosphoinositide 3‐kinase/protein kinase B signaling pathway (Kan et al., 2017; Liu, Liang, Zhou, & Liu, 2019). These subtle effects of UHRF1 downmodulation on RB cell proliferation and viability suggested that UHRF1 targeting alone may not be efficacious for RB treatment, and subsequently led to exploration for therapeutic applications in combination with other agents. A recent study uncovered that UHRF1 downmodulation significantly sensitizes RB cells to standard chemotherapeutic drugs such as etoposide by impairing DNA repair through downregulation of XRCC4 involved in nonhomologous end‐joining (NHEJ) repair. The decreased XRCC4 expression in UHRF1‐depleted cells reduces loading of DNA ligase IV onto damaged chromatin in response to etoposide treatment, resulting in defective DNA repair and higher apoptotic death (He, Lee, & Kim, 2018). Conversely, this study supports a model that enhanced DNA repair capacity driven by UHRF1‐mediated XRCC4 upregulation may protect RB cells against endogenous DNA damage‐induced cell death and thereby may promote outgrowth of malignant RB cells during tumor progression (Figure 2). Following this finding, another study demonstrated that UHRF1 depletion in RB cells can also augment sensitivity to histone deacetylase inhibitors (HDACi) by increasing oxidative stress‐mediated apoptosis via downregulation of redox‐responsive genes GSTA4 and TXN2. Consistent with the results from cell studies, UHRF1 depletion in RB cells increased the therapeutic efficacy of HDACi in murine orthotopic xenografts (Kim et al., 2020). Apart from augmentation of apoptosis in UHRF1‐depleted cells in response to HDACi treatment, the study also showed that UHRF1 downmodulation derepresses expression of photoreceptor‐specific genes in RB cells by decreasing HDAC association and concomitantly increasing histone H3 acetylation at the gene promoters, demonstrating that UHRF1 plays a role in epigenetic repression of differentiation‐associated genes in RB cells (Kim et al., 2020).

Taken together, UHRF1 downmodulation itself does not cause dramatic changes that would compromise RB cell proliferation and DNA methylation profiles but modulates the expression of various genes in cellular stress control to elicit more robust cell death when cells are challenged with other agents (Figure 2). This property of UHRF1 targeting may be conducive to development of efficient combination therapies as it would improve the efficacy and selectivity of conventional chemotherapy regimens composed of nontargeted drugs against tumor cells without a risk of complications involved in DNA methylation. As UHRF1 is not expressed in normal retina and nontumor retinal tissues in human RB, this would also provide another layer of selectivity for UHRF1 targeting in RB.

### 3.3 Enhancer of zeste homolog 2 and polycomb repressive complex 2

EZH2 is the catalytic subunit of polycomb repressive complex 2 (PRC2), which functions as a histone methyltransferase catalyzing di‐ and trimethylation of lysine 27 on histone H3 (H3K27me2/3) for transcriptional repression of target genes (Cao et al., 2002; Kuzmichev, Nishioka, Erdjument‐Bromage, Tempst, & Reinberg, 2002). The EZH2 is involved in the maintenance and differentiation of both normal embryonic stem cells and cancer stem cells (Richly, Aloia, & Di Croce, 2011). Overexpression of EZH2 has been documented in numerous cancers and shown to be associated with aggressiveness, metastasis, and overall poor outcomes (Bachmann et al., 2006; Simon & Lange, 2008; Takawa et al., 2011). Consistent with the role in development and differentiation, EZH2 is required for normal retinogenesis processes (Iida et al., 2015; Zhang et al., 2015), but its expression is subsequently silenced in postnatal retina after completion of retinal development (Khan et al., 2015). However, in human RB tissues, high EZH2 expression is maintained unlike normal retina (Ganguly & Shields, 2010; Kapatai et al., 2013; Khan et al., 2015). There are at least two different mechanisms accounting for the increased expression of EZH2 in RB tumors. As stated previously, EZH2 is a direct transcriptional target of E2F family proteins, which would be a critical driving force for EZH2 overexpression in tumors with *RB1* loss. Interestingly, increased EZH2 expression in other solid tumors has been associated with loss of EZH2‐targeting microRNAs such as miR‐101 and miR‐26A (Lu et al., 2011; Varambally et al., 2008). In this respect, miR‐101 was found to be downregulated in human RB tissues and experimentally shown to inhibit EZH2 expression by targeting its 3′UTR, providing a mechanistic link between EZH2 overexpression and miR‐101 downregulation in human RB tumors (Lei et al., 2014). To evaluate oncogenic roles of EZH2 in RB cells, pharmacological inhibition of EZH2 in human RB cells in comparison with primary fetal retinal pigment epithelium (RPE) cells was performed with two EZH2 inhibitors, GSK126 and SAH‐EZH2. The GSK126 is an S‐adenosylmethionine‐competitive catalytic EZH2 inhibitor blocking H3K27 methylation while SAH‐EZH2 is a hydrocarbon‐stapled peptide that disrupts the interaction between EZH2 and embryonic ectoderm development in PRC2 complexes which is required for EZH2 activity (McCabe & Creasy, 2014). Both types of inhibitors were effective in reducing RB cell viability whereas primary fetal RPE cells used as normal retinal cells in this study were not affected by these inhibitors (Khan et al., 2015). This finding suggests that EZH2 can be selectively targeted for RB treatment without significant effects on normal retinal cells, and also supports a rationale that lack of EZH2 expression in normal retina may confer selectivity for EZH2 targeting in RB patients. Another important point inferred from this study is that the similar anticancer activity of SAH‐EZH2 and GSK126 in RB cells may indicate that EZH2 would exert its oncogenic function mainly in the context of PRC2 complex in RB although non‐PRC2 or catalytic activity‐independent functions of EZH2 have been reported in other malignancies (Xu et al., 2012; Yan et al., 2013). Consistent with the oncogenic activity of PRC2 in RB, knockdown of suppressor of zeste 12 (SUZ12), another core subunit of PRC2, has been shown to inhibit RB cell invasion properties (Zhou, Sun, Liu, & Ma, 2014). Moreover, jumonji and AT‐rich interaction domain containing 2 (JARID2) which forms a stable complex with PRC2 and recruits the whole complex to the PRC2 target genes was found to be highly expressed in human RB tumors by two independent gene expression studies (Ganguly & Shields, 2010; Kapatai et al., 2013; Pasini et al., 2010).

### 3.4 B lymphoma Mo‐MLV insertion region 1

BMI1 proto‐oncogene is a polycomb group protein implicated in stem cell self‐renewal. Although BMI1 is required for normal maintenance of stem cells in diverse tissues, it can also induce tumorigenesis in the same tissues as signaling pathways promoting self‐renewal of stem cells can be adopted by cancer cells to facilitate their proliferation and survival (Pardal, Molofsky, He, & Morrison, 2005). As a component of polycomb repressive complex 1 involved in stable maintenance of gene silencing, BMI1 promotes self‐renewal of stem cells largely by repressing the expression of p16^INK4A^ and ARF tumor suppressors encoded from a single gene locus and thereby impairing p16/pRB and ARF/p53 pathways (Grinstein & Wernet, 2007; Schwartz & Pirrotta, 2013). Therefore, a tight balance between proto‐oncogenes such as BMI1 and tumor surveillance mechanisms is essential for maintaining stem cell functions throughout life without developing tumors by disabling any cells susceptible to oncogenic transformation. In this context, tumors may arise from mutated stem cells or restricted progenitors/differentiated cells that have acquired a self‐renewal capacity as a result of genetic or epigenetic aberrations. In the case of RB, the p16/pRB pathway is inherently inactivated and BMI1 overexpression can be driven by increased E2F activity as demonstrated by binding of E2F1 on *BMI1* promoter in human neuroblastoma cell lines (Nowak et al., 2006). Consistent with this, a gene expression profiling study on human RB identified BMI1 upregulation, and BMI1 immunohistochemistry on 34 archived human RB sections verified its wide expression (Kapatai et al., 2013; R. Ren et al., 2013). Of note, a high portion of BMI1‐expressing cells were confined to undifferentiated tumors and tumors with invasion to optic nerve and/or choroid, implying a role for BMI1 in RB progression to advanced tumor stages. Moreover, exogenous BMI1 expression in Y79 cells stimulated proliferation but inhibited apoptosis, with opposite effects observed with BMI1 depletion in Y79 cells (R. Ren et al., 2013). These results suggest that BMI1 may play a critical role in RB cell proliferation and tumor progression.

Functional investigations on *Bmi1* gene in mouse retinal development demonstrated that high Bmi1 expression can mark a rare subpopulation of immature retinal progenitor cells (RPC)/stem cells from the main RPC population at embryonic day 12.5 (E12.5), and overexpression of human BMI1 could convert the E12.5 mouse retinal cells possessing a limited proliferation capacity into stem cell‐like RPCs showing enhanced self‐renewal properties. Furthermore, RPC proliferation was reduced in the peripheral retina of *Bmi1* ^−/−^ fetus and newborn mice (Chatoo, Abdouh, Duparc, & Bernier, 2010). Although it was reported that tumorigenic retinal stem‐like cells can be identified from primary human RB tumors and these cells also have high BMI1 expression (Zhong et al., 2007), it would be of a particular interest to determine whether BMI1 is involved in RB stem‐like cell maintenance given the well‐defined function of BMI1 in stem cell biology. As cancer stem cells are believed to be responsible for tumor recurrence and therapy resistance, a better understanding of the role for BMI1 in RB would be critical for tumor management and relapse control.

### 3.5 HELLS

Chromatin remodeling complexes use an ATPase activity to mobilize nucleosomes, remove histones from DNA, and promote histone exchanges. This generally leads to transcriptional activation by increasing accessibility of gene promoters by transcription factors and other components in transcriptional machinery (Clapier & Cairns, 2009). However, chromatin remodeling activities can also promote transcriptional repression by facilitating chromatin binding of repressor complexes or increasing nucleosome occupancy in gene promoter and enhancer regions (Law et al., 2019; Ooi, Belyaev, Miyake, Wood, & Buckley, 2006).

Helicase, lymphoid specific (HELLS; also known as LSH, PASG, and SMARCA6) is a protein related to the SNF2 family of chromatin‐remodeling ATPases (Narlikar, Sundaramoorthy, & Owen‐Hughes, 2013). Previous studies demonstrated that HELLS is required for efficient maintenance of DNA methylation at repeat sequences in the mammalian genome presumably by associating with DNMTs (Dennis, Fan, Geiman, Yan, & Muegge, 2001; Myant & Stancheva, 2008), highlighting its contribution to the structure of constitutive heterochromatin. Moreover, the ATP‐binding site of HELLS was shown to be critical for de novo methylation at repeat elements by promoting stable association of DNMT3B, indicating that ATP‐dependent chromatin remodeling activity may be involved in global methylation regulation (J. Ren et al., 2015). Given that HELLS is frequently overexpressed in human cancers, these results have prompted several investigations for its role in cancer epigenome regulation. In hepatocellular carcinoma (HCC), HELLS overexpression has been shown to mediate epigenetic silencing of tumor suppressor genes by increasing nucleosomal occupancy at promoters and enhancers, thereby limiting the accessibility of the regions (Law et al., 2019). Interestingly, this study also revealed that there were no massive DNA methylation changes in *HELLS*‐knockout cells except for minor hypomethylation mostly at intergenic regions, despite the previous findings on the role of HELLS in DNA methylation. These results imply that HELLS mediates transcriptional repression through its chromatin‐remodeling activity but may not involve DNA methylation‐dependent mechanisms for gene silencing in HCC. Consistent with this observation, transcriptional repression by HELLS‐DNMT‐HDAC complex in reporter assays did not require enzymatic activities of DNMTs although both DNMT1 and DNMT3B were indispensable for association of HDAC whose activity was critical for HELLS‐mediated gene silencing (Myant & Stancheva, 2008). Therefore, HELLS appears to play a role in transcriptional regulation in cancer cells through at least two different mechanisms, functioning as a chromatin remodeler and/or a scaffold protein for recruiting other chromatin modifiers.

In RB, HELLS was initially identified along with UHRF1 as candidate genes contributing to tumor progression in murine RB models (Benavente et al., 2014). A recent study investigated the functions of *Hells* in retinal development and RB tumorigenesis using genetically engineered mouse models (Zocchi et al., 2020). *Hells* deficiency in retinal progenitor cells did not affect the overall development of retina; however, genetic disruption of *Hells* in *Rb1*/*Rbl1*‐double knockout mice led to a significant decrease in RB development with delayed tumor progression, supporting the notion that lack of timely transcriptional repression of *Hells* during retinal development owing to the loss of Rb family genes significantly contributes to RB tumorigenesis. As chromatin accessibility of flow‐sorted, *Rb1*/*Rbl1*/*Hells*‐triple knockout retinal cells was not significantly different from that of *Rb1*/*Rbl1*‐deficient cells, loss of *Hells* may reduce RB tumorigenesis through chromatin remodeling‐independent mechanisms. Instead, the study proposed that HELLS may serve as a transcriptional coactivator for expression of cell cycle genes, leading to proliferation of *Rb1*/*Rbl1*‐null retinal cells and consequent RB development (Zocchi et al., 2020). Although the mechanisms by which HELLS contributes to RB tumorigenesis still remain unclear, HELLS may be considered as a potential therapeutic target highly selective for RB cells as it is not expressed in terminally differentiated retinal cells and its inhibition does not affect normal retinal development.

### 3.6 High mobility group AT‐hook 2

High mobility group AT‐hook 2 (HMGA2; formerly known as HMGI‐C) belongs to the HMGA family of nonhistone chromatin proteins implicated in the assembly of multiprotein transcription complexes by directly binding to DNA at AT‐rich sequences. The HMGA proteins including HMGA2 are expressed at low or undetectable levels in adult tissues but are expressed highly in embryonic and neoplastic tissues (Fusco & Fedele, 2007). In support of the functional association of HMGA2 with cancers, pituitary adenomas develop in HMGA2‐transgenic mice and overexpression of HMGA2 induces neoplastic transformation in normal human lung cells whereas inhibiting HMGA2 expression suppresses the transformed phenotype in metastatic lung cancer cells (Di Cello et al., 2008; Fedele et al., 2002).

Consistent with the findings in other tissues, HMGA2 expression is high in murine embryonic retina and human RB tumors but is not detectable in terminally differentiated retina (Chau et al., 2003). Subsequent analyses of HMGA2 expression in large cohorts of primary RB tumors revealed that about 50–60% of cases show moderate to high expression of HMGA2 and a significant correlation exists between its expression and invasiveness of tumors (M. K. Singh et al., 2017; Venkatesan et al., 2009). Adenoviral antisense‐mediated blocking of HMGA2 synthesis inhibited RB cell proliferation, and microRNA profiling in HMGA2‐knockdown RB cells indicated that *miR‐106b~25* cluster may be a key downstream mediator for the oncogenic functions of HMGA2 in RB by modulating the expression of p21 and BIM (Chau et al., 2003; Venkatesan et al., 2014). Although several studies suggested positive roles for HMGA2 in RB cell proliferation, it remains unclear whether HMGA2 promotes cell proliferation by directly affecting E2F transcription factors and their target genes. A known mechanism by which HMGA2 promotes cell proliferation and pituitary tumorigenesis requires direct binding of HMGA2 to pRB, which counteracts pRB‐mediated inhibition of E2F1 activity by displacing HDAC1 from the pRB/E2F1 complex and facilitating acetylation of both histones and E2F1 protein at the target promoters (Fedele et al., 2006). As RB cells possess intrinsically upregulated E2F1 activity due to the loss of pRB, HMGA2 is likely to employ a different mechanism to promote RB cell proliferation. Alternatively, HMGA2 may directly interact with E2Fs and further enhance their transcriptional activity as HMGA proteins can stimulate DNA binding activity of transcription factors such as serum response factor by direct protein‐protein interactions (Chin et al., 1998). Aberrant expression of HMGA2 in human RB as opposed to normal retina suggests that HMGA2 expression is derepressed during RB tumorigenesis. Rearrangements in *HMGA2* gene leading to chimeric transcripts have been known to contribute to neoplastic transformation in mesenchymal tumors (Fusco & Fedele, 2007; Schoenmakers et al., 1995), but genetic alterations are not the cause of HMGA2 induction in RB and *RB1* inactivation does not seem to be a direct driver for HMGA2 expression as not all RB tumors and *RB1*‐null cancer cells express HMGA2. Instead, the deregulation of microRNAs appears to be associated with HMGA2 expression in RB, which implicates downregulation of let‐7 and miR‐98 in RB as a causal factor for HMGA2 induction (Li et al., 2019; Mu et al., 2010).

From the therapeutic perspective, a high correlation of HMGA2 with metastasis in poor prognostic cancers including RB may support its potential application as a biomarker in the blood or possibly aqueous humor since detection of HMGA2 mRNA in peripheral blood samples of patients with metastatic breast cancers has presented a reasonable expectation of success in other cancers (Langelotz et al., 2003). Interestingly, HMGA family proteins including HMGA2 have been suggested to impede DNA repair by downregulating the transcription of genes involved in various aspects of DNA repair processes in cancer cells (Reeves & Adair, 2005). Therefore, high expression of HMGA proteins may potentiate genotoxic stress induced by different DNA‐damaging agents and selective use of genotoxic agents for patients with high HMGA expression may be an attractive strategy to increase the therapeutic efficacy. However, RB tumors do not seem to have DNA repair defects despite high expression of HMGA2 and also HMGA1, as evidenced by the low mutation rates in the genome (Grobner et al., 2018; Kooi et al., 2016; Zhang et al., 2012). Rather, there are evidence indicating that RB tumors might have an enhanced DNA repair capacity than normal retina as genes involved in DNA repair/DNA damage pathways are among the top enriched gene categories upregulated in primary RB tumors from gene expression analyses (Ganguly & Shields, 2010; Kapatai et al., 2013). Moreover, UHRF1, which is highly elevated in RB has been experimentally shown to increase the expression of XRCC4, a critical factor for NHEJ repair (He et al., 2018). Therefore, the potential roles of HMGA2 and other HMGA proteins in chemosensitization of RB cells require further experimental validations. Of note, a stable phosphorothioate‐modified HMGA2 aptamer induced dose‐dependent cytotoxicity in RB cells while no significant cytotoxicity was detected in nonneoplastic human Müller glial cells (Nalini et al., 2016), which opens a possibility of therapeutic applications.

## 4 THERAPEUTIC IMPLICATIONS OF TARGETING EPIGENETIC REGULATORS IN RB

Epigenetic drugs targeting abnormal DNA methylation and histone acetylation have been efficacious for a subset of hematological malignancies (Duvic et al., 2007; Kaminskas, Farrell, Wang, Sridhara, & Pazdur, 2005). However, concerns for epigenetic drugs remain high regarding their potential off‐target effects beyond chromatin levels and selectivity for cancer cells, which may discourage clinical tests for these drugs in other cancers (Azad, Zahnow, Rudin, & Baylin, 2013). As chromatin modifiers are involved in gene regulation in both normal and cancer cell contexts, whether epigenetic drugs can selectively target cancer cells without causing significant damages to adjacent normal cells is an important question to be addressed before considering them as potential therapeutics. In this regard, targeting epigenetic regulators in RB provides a unique opportunity to selectively target only cancer cells. As stated earlier, many of these misregulated chromatin regulators in RB are not expressed in normal retinal tissues, conferring a selectivity for targeting. Furthermore, RB is a tumor where local therapies such as intra‐arterial and intravitreal chemotherapy can be safely performed in clinical settings (Wyse, Handa, Friedman, & Pearl, 2016; Yanik, Gunduz, Yavuz, Tacyildiz, & Unal, 2015). Localized drug delivery to affected eyes and absence of the molecular targets affected by the drugs in adjacent normal tissues are expected to provide unparalleled advantages to be considered for a viable therapeutic option if specificity and toxicity profiles of these drugs are in acceptable ranges for clinical trials. Another advantage of targeting epigenetic regulators is that many of such drugs may be used in combination with conventional genotoxic drugs to achieve better therapeutic outcomes or reduce nonspecific toxicity by being able to lower the doses of genotoxic drugs to elicit the same response to therapy (Juo et al., 2015). The possibility of the latter is of a particular importance as RB is a childhood cancer and use of conventional cytotoxic or genotoxic drugs may damage delicate organs in young children, resulting in multiple late effects later in their life. This aspect would constitute the major rationale behind the effort to develop effective targeted therapies with minimal late effects for children even when current chemotherapy regimens composed of conventional drugs are highly efficacious for saving eyes and lives upon early diagnosis and timely treatment.

Following the approval of DNMT and HDAC inhibitors for clinical use, several new classes of epigenetic drugs have advanced to clinical trials for a variety of malignancies (Genta, Pirosa, & Stathis, 2019). For example, EZH2 inhibitors such as tazemetostat have already shown favorable clinical activity in patients with relapsed or refractory lymphomas (Gulati, Beguelin, & Giulino‐Roth, 2018). Several different EZH2 inhibitors are currently evaluated for nonhematological malignancies as well in Phase I/II clinical trials (Gulati et al., 2018; McCabe & Creasy, 2014). As RB has EZH2 overexpression and pharmacological inhibition of EZH2 in human RB cells shows a clear anticancer activity, preclinical studies on EZH2 inhibitors in RB tumor models will be required to further strengthen a possibility of clinical trials for these compounds in RB treatment. Currently, UHRF1‐specific small‐molecule inhibitors are not available although a few natural compounds have been reported to affect the level of UHRF1 mRNA and protein (Ibrahim et al., 2018; Krifa, Leloup, Ghedira, Mousli, & Chekir‐Ghedira, 2014). A recent study showed that berberine, a botanical alkaloid, targets UHRF1 by binding UHRF1 directly in its tandem tudor domain‐plant homeodomain domain and induces UHRF1 degradation in multiple myeloma cells (Gu et al., 2020). Although berberine has many other binding targets in addition to UHRF1, it was the first report of any compound whose physical interaction with UHRF1 protein is demonstrated by multiple experimental approaches. As UHRF1 downmodulation can sensitize RB cells to conventional genotoxic drugs and HDAC inhibitors (He et al., 2018; Kim et al., 2020), development of potent and selective small‐molecule inhibitors of UHRF1 would be a promising direction to pursue in the future. Besides, UHRF1 overexpression is observed in numerous cancers as is the case with EZH2, which may enable us to apply what is learnt from RB's case to other UHRF1‐overexpressing cancers if UHRF1 inhibitors can be successfully developed. Similarly, targeted therapies for other epigenetic regulators discussed in this review may have a wide range of applications as they are also overexpressed in many cancers of different cellular origins. Since most epigenetic regulators are thought to be druggable (Ganesan, Arimondo, Rots, Jeronimo, & Berdasco, 2019), thorough functional validation and prioritization of molecular targets amenable to selective targeting would be the first step toward the development of new therapeutics for RB treatment.

## 5 CONCLUSION

As one of the childhood cancers arising from developing tissues, RB has a relatively stable genome, featuring a low mutation rate and a few recurrent chromosomal alterations related to somatic copy number changes. These observations have stimulated investigations on the implication of epigenetic mechanisms in RB development in recent years. As a result, we now have started to appreciate that a number of chromatin regulators are recurrently misregulated in RB and they may play crucial roles in diverting the gene regulatory network towards neoplastic transformation following *RB1* inactivation during retinogenesis. Despite the progress in our understanding on this subject, causal roles of these epigenetic regulators in controlling gene expression and cellular pathways in RB have not yet been clearly defined although a possibility of potential therapeutic applications has been emerging. Therefore, more comprehensive understanding of the biological functions of these chromatin regulators in the context of RB development will be required before designing new therapeutics targeting these epigenetic regulators. In RB, targeted therapy has not been in place yet as a common therapeutic modality in clinics. Ongoing efforts to further define the mechanisms of RB tumor progression including the contribution of epigenetic dysregulation to the molecular signature of RB will continue to unravel new therapeutic targets and potential biomarkers for prognosis.

## CONFLICT OF INTERESTS

The authors declare that there is no conflict of interests.
